# The Application of Massage in the Treatment of Dental Pathological Conditions

**Published:** 1891-02

**Authors:** W. F. Rehfuss


ARTICLE V.

### THE APPLICATION OF MASSAGE IN THE TREATMENT OF DENTAL PATHOLOGICAL CONDITIONS.

BY W. F. REHFUSS, D. D. S.

The utilization of massage in the treatment of various dental pathological conditions, is founded on its successful application, in general and special branches of medicine. In dentistry, alone, its therapeutic qualities have not been recognized; perhaps, because of the numerous difficulties that confront the dentist in the massage of the mucous membrane of the human mouth; the area being small in comparison with the leg or arm where the manipulations are easily performed. When in addition, we consider that the mucous membrane is more delicate than the epidermis of the leg or arm, it is evident that the dentist must exercise greater skill, and care in manipulating.

The term massage is applied to a series of systematic manipulations, which are best accomplished with the hands, such as kneading, friction, rolling or percussion of the external tissues of the body, the aim of which is to invigorate or restore to a healthy condition parts that may have become diseased or have lost their natural function.

The origin of massage can be traced almost to the Creation; as it was the first means resorted to by mankind for the relief of suffering. This is obvious, because almost every one instinctively seizes a part which is the seat of a sudden pain, and by rubbing or pressing endeavors to soothe it.

To the genius of Peter Henrik Link, of Sweden, credit is due for having instituted and placed upon a scientific basis, what are well known as the "Swedish movement cures." In 1813 the Royal Central Institution was established at Stockholm, with the patronage of the Swedish Government, that Link might practice and teach his system of gymnastics.

In France the country which gave massage its name and greatest impulses, this method of treatment has become neglected. The Germans and Scandinavians, however, have studied and practiced massage, with renewed zeal, and have thereby demonstrated its proper sphere of usefulness in medicine.

The Swedish method of manual treatment consists of different movements termed "Swedish movements." The movements are a form of calisthenics, the object of which is to exercise the natural function of a muscle, set of muscles, articulation, or limb, with the beneficial result of overcoming existing unnatural or pathological conditions. The movements are divided into passive and active. Massage is classed under the head of passive movements.

In adopting a system of applying massage, I have modified the methods of Dr. Mezger of Amsterdam, to conform to the special purposes of dentistry. He divides massage into four different manipulations; First, *effleurage* (centripetal stroking.) In general massage, this manipulation performed upon any portion of the body, as the leg or arm, consists of centripetal stroking (toward the heart.) This movement is similar to that of a carpenter's plane—a forward and backward motion and is performed with the palm of the hand, tips of the fingers or thumb.

Second, frictions, which are strong circular manipulations, given with the thumb or tips of the fingers.

Third, *petrissage* (kneading) is performed by the thumb or index finger. In general massage, an operator picks up a special tissue, muscle or tendon, and placing one finger on each side of the part, proceeds in centripetal motions with a firm pressure.

Fourth, *Tapotement* (percussion) which is divided into four kinds.

(a) *Clapping*, performed with the palms of the hand.

(b) *Hacking*, with the ulnar border of the hand.

(c) *Punctation*, with the tips of the finger.

(d) *Beating*, with the clenched fist.

Before commencing massage care must be observed in making an accurate diagnosis of the case; for upon that depends the nature of the manipulations, their force and frequency and the length of time during which they are to be employed.

In explaining the mode of performing dental massage, I will select a typical case, and describe the manipulations and *modus operandi* adopted. I will consider an acute pericementitis associated with pulpitis.

The first important point to be observed by the operator is that his position by the side of the patient, is easy and natural. In performing manipulations on the left side, the operator stands to the right of his patient and uses the thumb and fingers of the right hand for manipulating, and if necessary, the left hand for the application of a napkin. In manipulating the right side he stands to the left side of his patient using the fingers of his left hand.

The position of the hand and fingers is of much importance. The operator may use the fore finger or the middle finger, or both in manipulating, supporting the thumb on the adjacent teeth, as a pivotal or fixed point, that it may serve as a support and guide. The fore-finger of the other hand serves to hold up the corner of lip.

Again, the thumb may be used to perform massage, while the four fingers of the same hand rest on, or grasp the cheek.

Commence, with the introductory massage, which consists of *effleurage*—centripetal stroking,—around the affected part, and not *directly* over the seat of the inflammation, as thus the tendency would be to cause increased pain and inflammation. If we have a molar tooth, commence the stroking at the central incisors on the healthy tissue, using the thumb, fore, or middle finger for stroking. The stroking should be gentle at first, using scarcely any pressure. Continue this manipulation toward the affected tooth. The healthy tissue beyond should be similarily stroked.

This causes a soothing and anæsthetic effect, which enables us to approach gradually near the seat of the inflammation, causing less or even no pain at all, and the circulation is restored, so that exudations are carried off more easily. Carefully graduate the force of the manipulations, so as not to bruise the parts, but at the same time, a slight pressure must be used that the operator's fingers shall not slip.

Pain is relieved by the removal of the pressure from the terminal nerve filaments. To demonstrate this, it hardly needs the experiments of Glax and Klemensiewiez, who have proved that in an inflamed region, in consequence of the abnormal activity of the vascular walls the lymph spaces are so choked up by the exudations and products of inflammation poured into them, that their outlets become insufficient, and the efferent vessels are thus compressed and the circulation impeded. Therefore if we exert pressure upon an inflamed region already suffering under partial compression of its efferent vessels, it may injure rather than benefit.

We should manipulate cautiously until having gradually approached the seat of the disturbance, gentle stroking may be used directly over the pericementitis. Follow this by deep manipulations, gradually increasing the pressure, which can be considerable, without causing pain in a part that but a short time previous was very responsive to the touch.

When this stage of the operation has been reached, recourse to "frictions" can be had, starting from the anterior part of the mouth, and manipulating toward the posterior portion, using the fore-finger. After applying friction, we again use centripetal stroking, which is always used before and after frictions.

One of the diagnostic points, in cases of pericementitis, is that the tooth gives evidences of a sense of soreness on tapping it, seeming longer than the others.

In massage, I take advantage of this fact, and during the different stages of manipulations I test the tooth by tapping it with an instrument, and if the soreness becomes less, I know that the treatment is beneficial. In certain acute stages, the soreness disappears almost entirely when the manipulations are finished. After using stroking and frictions, *petrissage* (kneading) the third form of massage is applied. This manipulation consists of a series of small movements, performed by the fore-finger or thumb, and it differs from friction, because in the latter very little pressure is exerted, while *petrissage* consists of frictions with an additional pressure. However, I now use a modified form of kneading, discarding the circular movements, as I find at this stage of the manipulations, by using plain centripetal stroking and by giving an intermittent pressure, the same effect is produced more easily. The gum now has a resisting surface—slightly roughened similar to a piece of erasing rubber. By pressing the finger along the gum an intermittent jarring pressure is produced, similar to that felt by the finger on a piece of rubber, on which, without pressing, a resisting and not a sliding motion will result. Little, or in fact, no pressure should be used with this mode, as each resistance develops the succeeding pressure, the noise of which can be distinctly heard.

*Tapotement* (percussion) is the last manipulation used. Its application was a difficulty not easy to overcome, therefore I devised a small appliance for use in the dental engine, thus accomplishing the desired manipulation, which is a combination of clapping, hacking and beating. The appliance consists of a mandrel or shaft, to fit in the hand-piece of a dental engine. At the end is a small metal hub, and attached to it at right angles with the mandrel, by means of a cap screwing down, are three pieces of elastic French tubing, one-eighth of an inch in diameter, and one inch or one inch and a half in length—arranged like the spokes of a wheel radiating from the centre. When this appliance, the *percuteur*, is revolved in the hand-piece, their elasticity allows a succession of light blows to be given, the intensity of which is regulated by the speed of the engine.—*Dental Mirror*.

(*To be continued.*)

